# Burnout as a social pathology in nursing professionals: an analysis based on the theory of recognition

**DOI:** 10.47626/1679-4435-2022-771

**Published:** 2023-02-03

**Authors:** John Camilo Garcia

**Affiliations:** 1 Grupo de Investigación Salud Familiar y Comunitaria, Facultad de Ciencias de la Salud, Corporación Universitaria Remington, Medellín, Antioquia, Colombia; 2 Departamento de Bioética, Universidad El Bosque, Bogotá, Colombia

**Keywords:** burnout syndrome, social recognition, history of nursing, nursing care, social justice., síndrome de *burnout*, reconocimiento social, historia de enfermería, cuidado de enfermería, justicia social.

## Abstract

This is a reflexive article on burnout syndrome, founded on the theory of moral recognition from a historical and social framework, in order to design strategies to cope with burnout as a socio-cultural problem in the context of nursing. This theoretical reflection was developed using studies intentionally selected from the literature, especially the theories of recognition by Honnet and Fraser, and the historical analysis of nursing care proposed by Colliere. Burnout consists of a social pathology, its socio-historical factors connote lack of recognition of care and of nurses as such. This problem affects the shaping of a professional identity and leads to loss of the socioeconomic value of care. Therefore, in order to cope with burnout, it is necessary to achieve better recognition of care and of the nursing profession, not only from the economic point of view but also from the socio-cultural one, making nurses resume their social participation and free themselves from domination and disrespect, in order to contribute to social formation. Mutual recognition crosses the individuality of each subject, enabling communication with the others based on recognition of oneself.

## Abstract (es)

Este es un artículo de carácter reflexivo sobre el síndrome de *burnout*, planteado desde la teoría del reconocimiento moral, a través de un marco histórico-social, con el fin de esbozar estrategias para hacerle frente al *burnout* como problemática sociocultural en el ámbito de la enfermería. Para la elaboración de esta reflexión teórica, se utilizaron estudios de literatura seleccionados intencionalmente, en especial, las teorías del reconocimiento de Honnet y Fraser y el análisis histórico del cuidado de enfermería de Colliere. El *burnout* se configura como patología social, en la medida que factores sociohistóricos connotan una falta de reconocimiento del cuidado y del enfermero como tal, dicha problemática, afecta la configuración de una identidad profesional y lleva a una pérdida del valor socioeconómico del cuidado. Por tanto, para hacerle frente al *burnout*, es necesario un mejor reconocimiento no solo económico, sino sociocultural, del cuidado y de la profesión enfermera, haciendo que el enfermero retome su participación social y se libere de la dominación y del desprecio, con el propósito de aportar a la formación social. El reconocimiento mutuo atraviesa la individualidad de cada sujeto, posibilitando la comunicación con el otro desde el reconocimiento de sí.

## INTRODUCTION

Burnout syndrome was first described in 1960 in Graham Greene’s novel “A burnout case,” which popularized the term. However, it was only in 1974 that Freudenberger’s work^^1^^ described this syndrome as a condition associated with signs, symptoms, and etiological factors, consisting of a combination of demoralization, disillusionment, and exhaustion and being a specific hazard for naïve idealistic, young service professionals; hence, his work introduced a new description in which burnout is associated with negative stress and labor dysfunction.

As a set of signs and symptoms, burnout may be described as a condition based on the prolonged depletion of individual’s energies, and characterized by emotional exhaustion, reduced personal accomplishment, and feelings of insufficiency and depersonalization.^^2^^ This syndrome presents certain aspects and other characteristics that are related to the individual, always involving the context and the organization or way of working, all of them influenced by living conditions. In other words, burnout has multiple causes, and stressors usually derive from several factors that act simultaneously but differently.^^3^^

Among health care workers, nurses have been reported to present a higher prevalence of burnout.^^4^-^6^^ The frontline health care provided by these professionals makes them particularly susceptible to burnout, due to physical and psychological stress resulting from comprehensive patient care.^^7^^ The distressing mismatch between expectations and the reality of nursing profession increases propensity to burnout,^^8^^ leading to concerning disease rates among nurses. In a meta-analysis^^9^^ conducted prior to the coronavirus disease 2019 (COVID-19) pandemic, the prevalence of burnout was estimated at nearly 11.23%, whereas studies conducted during the pandemic reported rates from 30 to 50%,^^4^,^10^,^11^^ with a higher incidence in women than in men, even among professionals working in the same profession.

With regard to the causes of burnout, they could be grouped into global, institutional, and interpersonal causes.^^12^^ At the global level, burnout has been related to situations such as social inequality, unmet basic needs, lack of access to health services, differences between public and private institutions and between high- and low-income countries.^^11^^ However, another relatively new factor that has been added to increased workload in times of COVID-19 is social stigmatization of health care workers, associated with a feeling of non-recognition and lack of engagement from the rest of the community in the fight against the pandemic, which is associated even with aggressions and lack of social value of health care professions, as described by Cassiani-Miranda et al.^^13^^

At the institutional level, the main causes of burnout syndrome are related to work overload, shortage of personnel, lack of resources, and especially low economic recognition, whereas, at the interpersonal level, the main causes have been related to conflicts among professionals and problems in the professional-patient relationship.^^12^,^13^^ Therefore, in these three causal dimensions it is possible to identify factors related to non-recognition of the profession or to its low social value, low economic recognition of labor, and invisibility of care, up to a negative transvaluation of care as such.

Hence, a problem was identified, centered on the lack of recognition that underlies burnout syndrome, with a deep ethical and philosophical significance, such as the limitations imposed by the new ways of living, inexorably related to a historical frame that determines the self-accomplishment of a person. These conscious or unconscious limitations (which sometimes go unnoticed and tend to be reproduced) affect nursing professionals in the personal, institutional, and social domains. Thus, this study proposes an analysis of burnout based on the theory of recognition.

## METHODS

This is a reflexive theoretical essay developed intentionally using studies from the literature, covering the following descriptors: burnout, nursing, recognition.

This locus of investigation arises from a review on burnout among nursing professionals in times of pandemic, with emphasis on the understanding of burnout as an issue with multiple causes, one of which is lack of recognition, whose complexity requires a deconstructive analysis from the philosophy of knowledge, based on Axel Honnet and Nancy Fraser, theorists of recognition, and on Marie Colliere for analysis of care. Therefore, a study aiming to reflect upon burnout and recognition was conducted from January to February 2021.

Additionally, a search for relevant information on the theme of the study was conducted using the following databases: Latin American and Caribbean Health Sciences Literature (*Literatura Latinoamericana y del Caribe en Ciencias de la Salud*, LILACS), Virtual Health Library (Biblioteca Virtual en Salud, BVS), Scientific Electronic Library Online (SciELO).

Since this research does not involve human beings, the need for an opinion from a research ethics committee was waived.

## RESULTS

### INVISIBLE CARE FROM COLLIERE TO HONNETH

According to Colliere,^^14^^ invisibility of care, lack of social value, and thus economic devaluation of nursing care have deep historical and social roots, starting with the confiscation of women’s knowledge of the use of writing, held in men’s hands. Furthermore, the development of an ideology of care based upon dedication to the poor and salvation of the soul took the place of a fundamental knowledge of body care practices. This paved the way during the nineteenth century for the increased value of technical cure and consideration of care as a menial work, worthless, requiring no ability, no knowledge, and therefore socially and economically unrecognized.^^14^^

Although social, professional, and family dynamics have considerably changed, including one-parent families and increased number of professional nurses,^^15^^ most social and economic protection systems are still marked by ideological connotations that do not consider the profound social change resulting from the deconstruction of the gender system and the consequent symmetry of roles that is about to inevitably occur. For this reason, the model based on the ancient social conception founded on the sexual division of labor reluctantly persists, assigning different tasks, roles, and behaviors to men and women.^^16^^

Nursing care, as well as the core of the nursing profession, is strongly related to female activities, such as food preparation, children’s education, or household chores; even nowadays a connotation of intrinsic naturalness is perceived to be associated with gender, losing the reciprocity of activities and making it difficult to insert them into a system of change. In other words, a persistent erroneous conception normalizes and naturalizes these activities from a derogatory perspective of gender, in which they no require effort (are natural) nor have any social value, and thus are exchangeable in a system of market. Therefore, treating a patient or applying an injection is usually regarded as a natural favor, in contrast to other tasks genealogically associated with the male gender. According to Colliere,^^17^^ the impact of this cultural past generates loss of knowledge, associated with inequality in the sexual division of labor, as well as the influence of religious values conveyed since the Middle Ages. Hence, there is a slow but certain economic devaluation of care measures provided by women.

Invisibility occurs both in interpersonal and social domains, but in an analog way, is in the interpersonal domain that individuals may ascertain their own social visibility based on the fact that other people ensure their existence. This means, according to Honneth,^^18^^ as an inverse conclusion, that only the absence of such reactions may establish one’s “invisibility”. From the perspective of the affected individual, the criterion used to ensure their visibility, in the figurative sense, is the externalization of determined reactions that are a sign, an expression that the individual is positively taken into account; thus, the suppression of such forms of expression indicates that, in this particular sense, an individual is not socially visible to the person in from of them.

“Making someone visible” goes beyond the cognitive act of individual identification and is manifested, in an evident manner, through corresponding actions, gestures, or mime, that the person is being favorably taken into account, according to the existing relationship; and only because we have a common knowledge of these emphatic forms of expression in the scope of our second nature, their suppression may be seen as a sign of invisibility and humiliation.^^19^^

Since care is an interdependent action, an action for the others, with the others, and of the others, it is an ecstatic action that goes beyond the self but that, at the same time, also goes beyond the cognitive act, of the nurse-patient duality, because their implications bring family, social, and even economic effects. However, such effects are mostly unknown by individuals unrelated to the profession or tend to be minimized by current financial health care models, which obliterate nurses’ contribution; therefore, the relationship between cost and quality of nursing care remains unknown.^^20^^

### DEVALUATION OF CARE: A NIHILISTIC PERSPECTIVE

Invisible, devalued care, but that aims to be recognized in a mercantilist world, faces a paradox: how to make care visible without losing its essence? How to deliver care in a world that only values what provides immediate benefits? In the current economy, which sees consumption and expenditure as positive and saving as a moral flow associated with meanness, is it necessary to frame health care as an action that generates profit or that can be purchased or sold?

Current society considers health as a commodity and understands health as well-being, but not simply well-being, rather as a state of perfect wellbeing. Health understood as well-being is an economically useful right, founded on the Keynesian model. The concept of health has been expanded almost up to the boundaries of human desires, reaching the utopia of a perfect state of fulfillment or happiness. From this perspective, life has been medicalized, and any medical condition, illness or distress has been pharmaceuticalized, always within the framework of profitability.^^21^^ Furthermore, one of the problems of medicalization of life and of health as a commodity is the fact that this phenomenon has no ending point, since the consumption of health may be increasingly encouraged, and boundaries are established by money rather than by time,^^21^^ as a cycle dependent on money in a world codependent of it, this relationship seems to have no end. Under this hegemonic model, care seems not to fit easily, since it is not an active part of pharmaceuticalization of life and nor can be measured or economically valued, which is why nursing is still deemed as a subordinate profession.

Care as a nursing value has undergone devaluation. In the dawn of humanity, care was one of the supreme values, being recognized and valued; however, it has lost that recognition, and nihilism of care, a history of devaluation of care work. In times of lack of sense, neither artistic men nor free spirits are recognized,^^22^^ which are the ones that, from Nietzsche’s perspective, turn care into an artistry. Overcoming devaluation requires the transvaluation of care, in order to understand it in its broader sense, addressing care for life in its full meaning, i.e., ontological and axiological care. People care for what they value, and they value that place or time when they were happy; hence, when could a person be happy if not when they are alive? Therefore, it could be stated that care coexists in Nietzsche’s myth of the eternal return, but in this care has not achieved freedom yet, it is still has the spirit of the camel, carrying all the burden, walking slowly, and being subjugated by others in its evolution.^^23^^ Likewise, nursing professionals bear with the majority of responsibilities, seem to walk slowly, and are dominated by others. In an occupation in which experience and academic training is little valued,^^24^^ one should not look outwards, but rather look inwards, i.e., towards the genealogy and phenomenology of care. However, in this path towards transvaluation, one suffers not only as a professional group but also as an individual, hence the importance of recognizing, in a context when health and wellbeing are seen as comparable concepts, this occupational and individual distress as a social pathology, which is now medicalized, classified as burnout syndrome, and neglected from the nursing point of view.

### BURNOUT AS A SOCIAL PATHOLOGY

Recognizing burnout as a social disease implies assuming the arguments of social philosophy that state how need for prestige and distinction are consequences of loss of freedom, social individualization, political apathy, and economic pauperization.^^18^^ However, what is regarded as a social defect involves not only matters of violation of justice principles but also all situations that restrict the life possibilities that are assumed as normal. If some conceptual categories from social philosophy are named social pathologies, including reification of individuals, loss of community, depersonalization, Disenchantment, and collective neurosis, would burnout be the result of a social pathology, or is it a social pathology by itself?

According to Han,^^25^^ there was the change from the disciplinary society described by Foucault to the performance society, going from duty to ability. In the twenty-first century, all is possible, everything can be solved, mended, changed, or improved; thus, why to care for? At least this is the reproduced paradigm in which care seems to be a barrier. In the context of this mismatch of care, nurses not only are in conflict with the dominant paradigm but also have to keep struggling. It is a functional mismatch, because nurses belong to a performance society that does not recognize their labor and tends to limit or subordinate it. The problem is not limited to this issue, since it is more difficult for health care professions to subvert the prevailing order without entering into conflict with professional ethics and with the duty of care.

Social organization of social labor is related to a system of social valuation of the labor, which plays a major role in the structure of knowledge of a society, establishing the cultural definition of the hierarchy of action tasks, of social appreciation that an individual may receive due to their activity, and of the characteristics linked to this activity. The possibilities of individual identity formation are directly related to the institutionalization and social distribution of labor, through the experience of recognition. Therefore, social and institutional recognition represents not only individual recognition, but also the very personal and professional identity. Human integrity depends on an experience of intersubjective recognition; thus, humiliation, and personal damage affect human dignity and disrespect and moral offense preclude individual and collective possibilities of life and, thus, hamper the moral progress of society. Hence, a disorder such as burnout, which is associated with the economic model and also with the history of the profession and with the paradigmatic conflict between the essence of care and economic interests, consists of a social pathology.

### RECOGNIZING FOR REDISTRIBUTING, OR REDISTRIBUTING FOR RECOGNIZING

Honneth formulated a theory of reciprocal recognition, based on three sources: Hegel’s idea of struggle for recognition, Foucault’s theory on struggle for power, and Habermas’ theory. Honneth recovers three Hegel’s theses: the basic premise of recognition, the intersubjectivity, according to which the constitution of individual subjective identity presumes reciprocal recognition and formation of the self, the latter is related to reciprocal recognition between subjects. Individuals can reach an understanding of themselves as they shape an individualized and autonomously active self only if they have their identity confirmed between each other.

The second thesis alludes to the fact that, in modern societies, there are different forms of recognition that are distinguished by the degree of individual autonomy (love, law, and solidarity). The third thesis, in turn, proposes that logic is a formative process mediated by the stages of moral struggle. Therefore, throughout the formation of their identity, subjects are obliged to, at each stage of socialization, enter into an intersubjective conflict, whose result is the recognition of their claims to autonomy not affirmed previously. Thus, individuals struggle not only for power and for material issues, but also for moral issues, which in turn result in struggles for power.

Recognizing burnout as a social pathology enables to better explore all factors associated with this disorder and makes it possible to take measures about the matter, since its onset is perceived to be related to a form of disrespect, subordination, and lack of recognition, which generates a mismatch in the current model. Hence, the perception of the forms of disrespect may motivate individuals to enter into a practical struggle or a conflict; to this end, it is enough to follow the thread of affective feelings that are associated with forms of disrespect to determine which modality of recognition is denied.

According to Honneth,^^18^^ three modalities of disrespect may be identified, each one denying one of the three spheres of recognition (love, law, and achievement): in the sphere of recognition encompassing love, the *principle of need* prevails; in the relationships established according to law and rights, the *principle of equality* will have priority; and in the sphere of achievement and in cooperative relationships, priority will be given to the *principle of merit.* If these relationships are positively developed, each one will have a result: in the first sphere, the accomplishment of *self-confidence*; in the second sphere, *self-respect*; and, in the third sphere, *social appreciation and self-esteem*.^^26^^ These spheres are not isolated; on the contrary, they are closely related, so that an impairment in one may have an impact on the others.

In the nursing context, as previously shown, there is a lack of recognition of social labor (merit) and there is inequality in relation to other professions, as well as within the same profession according to geographical area and gender. Moreover, in the sphere of love, there is an unreciprocated affection that does not allow for the establishment of a strong professional identity in many professionals, considering that the sphere of love is not strictly circumscribed to the family environment but also includes most intimate personal relationships, such as relationship with coworkers, health care professionals, and patients. Therefore, the forms of disrespect associated with lack of recognition and that favor burnout syndrome in nurses are the following, according to Honneth: stigmatization, lack of professional identity (disrespect to merit), exclusion and marginalization (lack of equality), insecurity, and suspicion, findings similar to those observed in the studies by Kapu et al., McAndrew & Hardin, and Monsó Fernández et al.^^12^,^27^,^28^^

Burnout is an issue of social justice with characteristics of redistribution and recognition, i.e., of economic justice and historical-cultural justice. In order to cope with burnout, an integrative view is required, such as that proposed by Fraser & Honneth,^^29^^ since social recognition goes hand in hand with economic recognition, being consubstantial. In this sense, a theory of the struggle for moral recognition would result in a better economic recognition and vice-versa, but one cannot be achieved without the other.

As previously shown, lack of moral recognition of nursing professionals and of care, in the current context, is translated into economic injustice. Therefore, the first efforts should be directed to moral recognition, while redistributive efforts are explored, without losing the essence of care, since care represents, as a way of being in the world, the externalized way of living. Care for the others faces a mismatch in a world marked by individualism, and this should be precisely acknowledged as a difference; hence, it is worth recognizing the value that care can produce to the current world, which is the difference amidst the surrounding homogeneity. This difference is even more necessary in the performance and consumption society, a society that wastes not only the resources, but also the bodies.

Transvaluation of care should not lose its essence, and one should resort to professional ambidexterity as a way to handle the paradox of valuing care in a market society. It is necessary first to understand how current society works and, second, what one’s strengths and weaknesses are, both in the individual and professional scopes, in light of one’s social, professional, and individual needs. Ambidexterity has been mentioned in the corporate scope as the ability to exploit the capabilities and skills provided by the context, while simultaneously exploring other methods that enable to improve intrinsic capabilities or acquiring new ones.^^30^^ Therefore, the paradox is faced by capturing the two extremes and integrating them.

Interdisciplinarity and critical thinking are required from individuals working in different worlds of thinking. Today, more than ever, this is required from philosopher nurses, clinical nurses, community nurses, administrative nurses, among others. It is necessary to transcend cognitive limits, as well as unifying and aggregating the different within the similar, in order to face a common problem through an ethically motivated theory, such as the one that understands recognition as a condition required for social interaction in which individuals who recognize or affirm each other in their faculties and qualities while knowing and recognizing the parts of their irreplaceable identity, which will enable them to oppose themselves to others. In its full meaning, identity is a moral principle, the principle that requires treat the others as worthy of respect and recognition.

## CONCLUSION

The aim of recognition is enabling nurses to resume their social participation and free themselves from domination, maintaining the purpose of social construction. Mutual recognition crosses the individuality of each subject, enabling communication with other from recognition of oneself. This inclusion of others is crossed by language, gestures, and communication, as individuals are able to take initiative from which freedom is manifested. It does not show what they are, but rather who they are, which occurs when one enters into contact with others and is recognized in a determined social space. Recognition of care enables to recognize nursing labor in the personal, institutional, and social domains. Therefore, solving the problem of lack of recognition makes it possible to face adversities, resist, persist, and do not lose heat, preventing depersonalization and emotional exhaustion, and thus burnout syndrome, which affects professionals’ health and quality of health care.
